# Global research trends of World Health Organization’s top eight emerging pathogens

**DOI:** 10.1186/s12992-017-0233-9

**Published:** 2017-02-08

**Authors:** Waleed M. Sweileh

**Affiliations:** Department of Physiology and Pharmacology/Toxicology, College of Medicine and Health Sciences, An-Najah National University, Nablus, Palestine

**Keywords:** Bibliometrics, Outbreaks, Virus, WHO, VOSviewer, AcrGIS 10.1

## Abstract

**Background:**

On December 8^th^, 2015, World Health Organization published a priority list of eight pathogens expected to cause severe outbreaks in the near future. To better understand global research trends and characteristics of publications on these emerging pathogens, we carried out this bibliometric study hoping to contribute to global awareness and preparedness toward this topic.

**Method:**

Scopus database was searched for the following pathogens/infectious diseases: Ebola, Marburg, Lassa, Rift valley, Crimean-Congo, Nipah, Middle Eastern Respiratory Syndrome (MERS), and Severe Respiratory Acute Syndrome (SARS). Retrieved articles were analyzed to obtain standard bibliometric indicators.

**Results:**

A total of 8619 journal articles were retrieved. Authors from 154 different countries contributed to publishing these articles. Two peaks of publications, an early one for SARS and a late one for Ebola, were observed. Retrieved articles received a total of 221,606 citations with a mean ± standard deviation of 25.7 ± 65.4 citations per article and an *h*-index of 173. International collaboration was as high as 86.9%. The *Centers for Disease Control and Prevention* had the highest share (344; 5.0%) followed by the *University of Hong Kong* with 305 (4.5%). The top leading journal was *Journal of Virology* with 572 (6.6%) articles while *Feldmann, Heinz R*. was the most productive researcher with 197 (2.3%) articles. China ranked first on SARS, Turkey ranked first on Crimean-Congo fever, while the United States of America ranked first on the remaining six diseases. Of retrieved articles, 472 (5.5%) were on vaccine – related research with Ebola vaccine being most studied.

**Conclusion:**

Number of publications on studied pathogens showed sudden dramatic rise in the past two decades representing severe global outbreaks. Contribution of a large number of different countries and the relatively high *h*-index are indicative of how international collaboration can create common health agenda among distant different countries.

## Background

On December 8^th^, 2015, World Health Organization (WHO) led a meeting of experts and health consultants in Geneva to discuss and publish a priority list of pathogens likely to cause serious outbreaks in the near future bearing in mind that the suggested pathogens had limited or no available effective therapies or preventive measures [1]. The meeting came up with a list of top eight emerging serious pathogens that are of great harmful health consequences. According to WHO, the list is not an ultimate one and is supposed to be reviewed annually to include any new emerging pathogens. The WHO list aims to lay the basis and background for national and international health planning to combat and control any potential outbreaks of these pathogens. Furthermore, the WHO wanted countries, researchers, clinicians, and policy makers to talk about these pathogens and corresponding infectious diseases as part of global awareness and preventive policies which might include developing new and inexpensive diagnostics, therapies, vaccines, and behavioral health measures.

According to WHO, the list of pathogens, which required urgent attention for research and development pertaining to preparedness, included “Crimean Congo haemorrhagic fever, Ebola virus, Marburg, Lassa fever, Middle East respiratory syndrome (MERS) and Severe acute respiratory syndrome (SARS) coronavirus diseases, Nipah, and Rift Valley fever” [1]. These infectious diseases are caused by viruses and some of them, such as Crimean-Congo and Ebola, are associated with high fatality rate [2–8]. Marburg virus is transmitted to people from fruit bats and spreads among humans through human-to-human transmission [9–13] while Lassa fever is transmitted to humans through food contaminated with rodent feces or urine [14, 15]. Middle East respiratory syndrome is caused by a coronavirus that was first identified in Saudi Arabia in 2012 [16–18] while SARS, another coronavirus respiratory disease, was recognized on February 2003 [19, 20]. Nipah virus, identified in 1998, is emerging zoonosis that affects both animals and humans [13, 21–24]. Rift Valley fever is a viral zoonosis that was first identified among sheep on a farm in the Rift Valley of Kenya [25–29]. The WHO committee listed another three pathogens/infectious diseases and considered them as serious and require an action as soon as possible. These three serious diseases include Chikungunya, severe fever with thrombocytopenia syndrome, and Zika.

Literature review using Pubmed, Google Scholar and Scopus showed that bibliometric studies on SARS or Ebola or Nipah virus have been carried out, but as a single disease and not as a group of diseases with potential future severe epidemics [25–29]. The collective analysis of literature on top eight pathogens will give a more comprehensive view on these infectious diseases and will help identify which one needs to be given top priority for funding and research.

It has been reported that mapping literature with certain statistical methods could help in detection of emerging infectious disease outbreaks particularly in the presence of internet with thousands of reports being easily communicated among public health specialists and healthcare providers [30, 31]. Based on all of the above, we carried out this bibliometric study to analyze literature on top eight emerging pathogens suggested by WHO. Specifically, information regarding number of publications over time, contribution of various countries, international collaboration, active authors and institutions, journals that are actively publishing articles, citations analysis, geographical distribution of publications, visualization of inter-country collaboration, and top cited articles will be presented. This kind of analysis will be of value to virologists, pharmacist, medicinal chemist, and clinicians who are interested in infectious viral diseases and in developing effective preventive and curative pharmaceutical products. Young researchers need to direct their research efforts toward emerging diseases because they are considered top priority and a bulk of financial support will be invested in these diseases. Healthcare workers in the field of travel medicine need to be aware of the map of infectious diseases that quickly cross borders from one country to another leading to spread of diseases with potential negative impact on public health and tourism industry.

## Methods

For this study, Scopus search engine was chosen to retrieve required literature. Scopus was used because of its advantages over other databases such as Web of Science (WoS), Google scholar or Pubmed [32]. According to Falagas et al. study, no database is perfect and each has certain merits over the other. For example, PubMed and Google Scholar are free to use in contrast to Scopus and WoS. PubMed lacks citation analysis in contrast to other databases. Scopus offers about 20% more coverage than Web of Science and 100% of Medline database is covered by Scopus. Google Scholar is the largest in terms of coverage but results obtained by Google Scholar have inconsistent accuracy. Although Scopus covers a wider journal range, it is currently limited to articles published after 1995 when compared with WoS [32]. In the current study, we preferred the use of Scopus because of its wider coverage since we are interested in global research activity in the eight emerging pathogens. Many of the journals published from developing countries, where these infectious diseases were found, are indexed in Scopus. This is reflected in the number of journals covered by Scopus versus those covered by WoS [32].

**Fig. 1:**
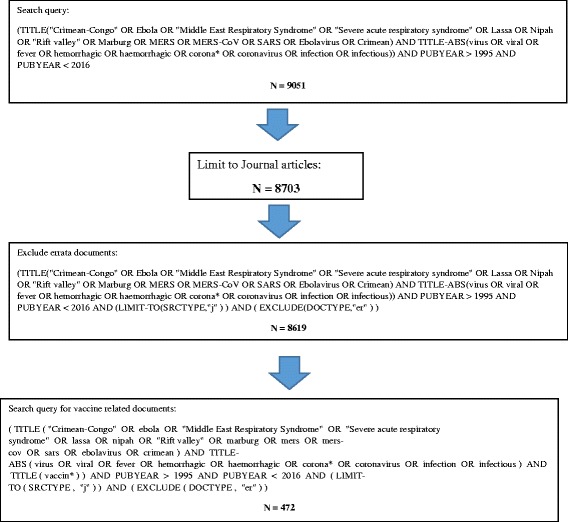
Strategy and search query used to retrieve required data in Scopus

In the current study, keywords used were the names of diseases that appeared in the WHO top eight list. To avoid errors, the names of diseases were followed by conditional keywords such as “virus OR viral OR fever OR hemorrhagic OR haemorrhagic OR corona* OR coronavirus OR infection OR infectious). Fig. 1 illustrates the steps followed along with keywords and search query used in Scopus to retrieve required data.

The data obtained were refined using the side functions in Scopus. Such functions include: 1) time limitation which was set for this study from 1996 to 2015, 2) source type of data which was set in this study to be journal articles while books and book chapters were excluded, and finally 3) type of documents and for the purpose of this study all types of documents were included except errata (correction).

Analysis of data was carried out using the “analyze” function in Scopus menu bar. Analysis included annual number of published documents, productivity of each country, author, preferred journals for publishing research on top eight emerging pathogens, geographical distribution, network visualization, and institution/organization. Scopus allows for citation analysis such as total number of citations, Hirsch index (*h*-index), and top cited articles. The *h*-index is a parameter used to measure productivity and scientific impact of an author, institution, or country, or even a subject area [33]. Scopus can also give analysis about active journals in publishing articles on studied diseases. Active journals were presented along with Impact Factor (IF) which was obtained from the Journal Citation Report published by Thomson Reuters.

An important feature in Scopus is that it allows exclusion or limitation which allow researchers to identify articles published by a single author or a single country. Based on this, we divided articles into two types: (1) single country publications (SCP) in which all authors have the same country affiliation and such publications represent an intra-country collaboration, and (2) multiple country publications (MCP) in which authors have different country affiliation and such publications represent inter-country collaboration.

In bibliometric studies, not all data can be presented. In most bibliometric studies, active or most productive countries, authors, institutions/organizations, and journals are usually presented. In this study, with large number of retrieved documents, only countries, authors, institutions, and journals with a minimum productivity of 100 documents were presented and ranked. The cutoff point of 100 publications have been previously used in other bibliometric studies [34]. For analysis pertaining to each infectious disease, only the top 10 productive countries were presented.

An important preventive aspect of most serious infectious diseases is the development of vaccines for prevention of spread. In this study, publications pertaining to vaccine development against any one of the top eight emerging pathogens were sought and presented. The search query used to search for vaccine development was the same search query used to retrieve publications on the top eight pathogens plus the keyword “vaccin*” with an asterisk to retrieve words such as vaccine or vaccination. The complete search query for vaccine data was presented in Fig. 1.

Statistical Package for Social Sciences (SPSS - 21) was used to create graphs pertaining to growth of publications for each disease. Mean ± standard deviation (SD) and median (Q1 – Q3) were used for descriptive statistics. Finally, bibliometric studies do not involve human or animal subjects and therefore, no ethical approval by Institutional Review Board was required.

## Results

**Fig. 2:**
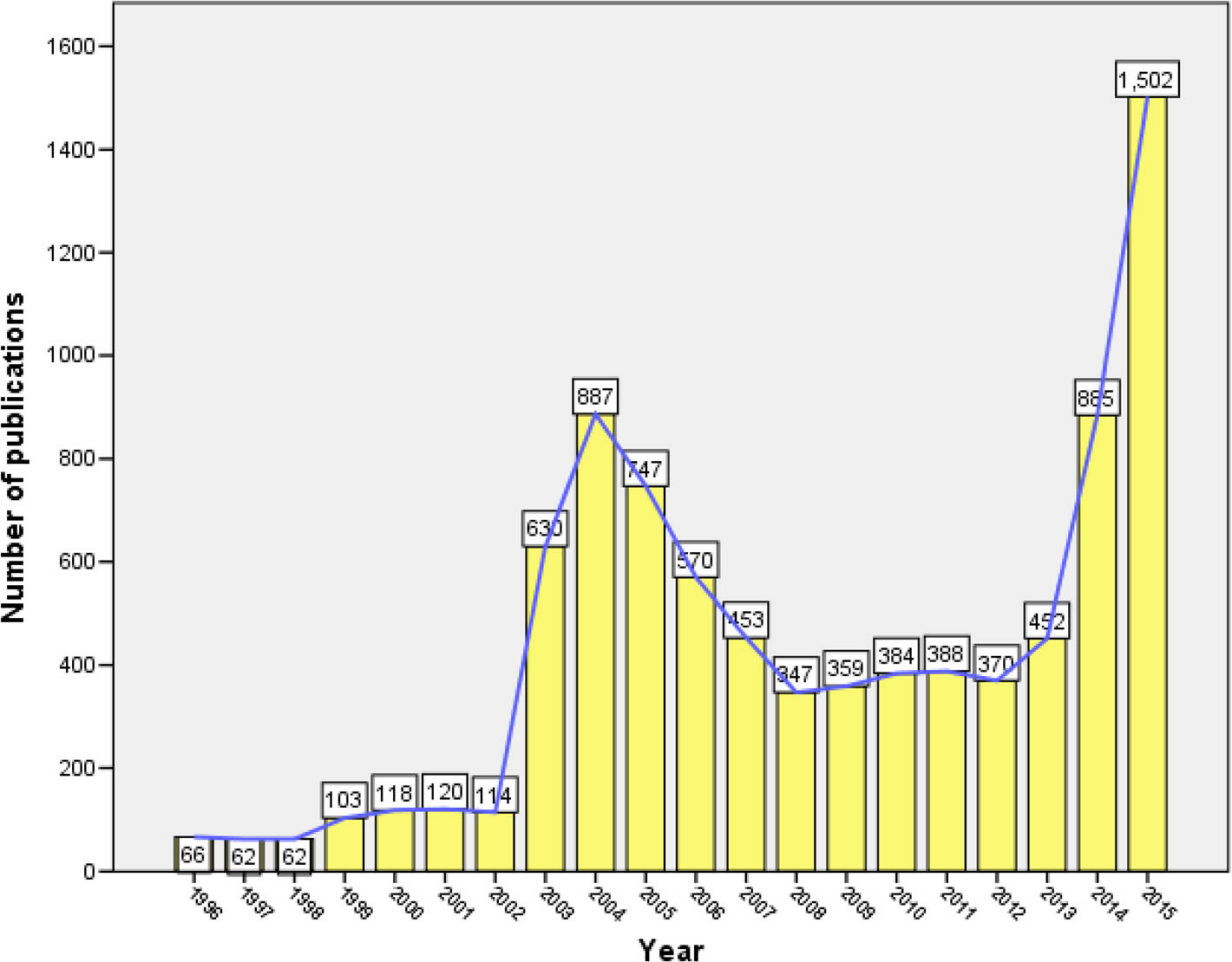
Annual growth of publications over the study period (1996–2015)

A total of 8619 journal articles were retrieved. The highest number of published articles was recorded in 2015 while the lowest number of published articles was recorded in 1996. The growth of publications showed a rising trend in 2003 and 2004 and then in 2014 and 2015. Figure 2 shows the annual growth of publications during the study period.

A total of 28 different languages were encountered in retrieved articles. English (*n* = 7,661; 88.9%) was the most common followed by Chinese (*n* = 387; 4.5%), French (206; 2.4%), and Russian (*n* = 131; 1.5%). The majority of retrieved articles were research articles (*n* = 6,587; 76.4%). Other types of retrieved documents are shown in Table 1.

**Table 1:** Types of retrieved documents

Type of document	Frequency	% *N* = 8619
Article	6633	77.0
Review	984	11.4
Letter	304	3.5
Note	258	3.0
Conference Paper	192	2.2
Editorial	141	1.6
Short Survey	107	1.2

The majority of articles (*n* = 5,406; 62.7%) were published in peer reviewed journals in the subject area of “Medicine” while 3075 (35.7%) were published in peer reviewed journals in the subject area of “Immunology and Microbiology”. The subject areas with a minimum of 100 articles are shown in Table 2. Since some journals fit into more than one subject area, the total percentages in Table 2 exceeded 100%.

**Table 2:** Subject areas of retrieved documents

Subject area	Frequency	% *N* = 8619 ^a^
Medicine	5406	62.7
Immunology and Microbiology	3075	35.7
Biochemistry, Genetics and Molecular Biology	1681	19.5
Pharmacology, Toxicology and Pharmaceutics	533	6.2
Agricultural and Biological Sciences	407	4.7
Veterinary	283	3.3
Multidisciplinary	234	2.7
Social Sciences	179	2.1
Chemistry	170	2.0
Environmental Science	133	1.5
Nursing	123	1.4

^a^Due to overlap among subject areas, the total percentages exceeded 100%

### Citation analysis

Retrieved documents received a total of 221,606 citations. The mean ± SD was 25.7 ± 65.4 citations per documents while the median (Q1 – Q3) was 9 (2–27). The *h*-index was 173. A total of 7291 (84.6%) articles were cited at least once while 1328 (15.4%) articles were not cited at all. A total of 408 (4.7%) publications received a minimum of 100 citations per article.

The article that received the highest number of citations was “*A novel coronavirus associated with severe acute respiratory syndrome*” [35] published in *New England Journal of Medicine* (*NEJM*) in 2003. It received a total of 1979 citations. Table 3 shows the top 20 cited articles. Content analysis of top cited articles showed that 18 articles were about SARS, one about Nipah virus and one about Ebola virus. Five of top cited articles were published in *NEJM*, three in *Lancet*, six in *Science*, and three in *Nature*.

**Table 3:** Top cited 20 articles on top eight emerging pathogens/infectious diseases

Article	Year	Journal	Number of citations
A novel coronavirus associated with severe acute respiratory syndrome [35]	2003	New England Journal of Medicine	1979
Identification of a novel coronavirus in patients with severe acute respiratory syndrome [93]	2003	New England Journal of Medicine	1810
Coronavirus as a possible cause of severe acute respiratory syndrome [94]	2003	Lancet	1535
Characterization of a novel coronavirus associated with severe acute respiratory syndrome [95]	2003	Science	1479
The genome sequence of the SARS-associated coronavirus [96]	2003	Science	1295
A major outbreak of severe acute respiratory syndrome in Hong Kong [97]	2003	New England Journal of Medicine	1135
Angiotensin-converting enzyme 2 is a functional receptor for the SARS coronavirus [98]	2003	Nature	943
Clinical progression and viral load in a community outbreak of coronavirus-associated SARS pneumonia: A prospective study [99]	2003	Lancet	916
Isolation and characterization of viruses related to the SARS coronavirus from animals in Southern China [99]	2003	Science	895
Identification of severe acute respiratory syndrome in Canada [100]	2003	New England Journal of Medicine	827
Bats are natural reservoirs of SARS-like coronaviruses [101]	2005	Science	720
A cluster of cases of severe acute respiratory syndrome in Hong Kong [102]	2003	New England Journal of Medicine	677
Unique and conserved features of genome and proteome of SARS-coronavirus, an early split-off from the coronavirus group 2 lineage [103]	2003	Journal of Molecular Biology	638
Fruit bats as reservoirs of Ebola virus [104]	2005	Nature	606
Nipah virus: A recently emergent deadly paramyxovirus [105]	2000	Science	605
Clinical Features and Short-term Outcomes of 144 Patients with SARS in the Greater Toronto Area [106]	2003	Journal of the American Medical Association	603
Severe acute respiratory syndrome coronavirus-like virus in Chinese horseshoe bats [107]	2005	PNAS^a^	568
Koch’s postulates fulfilled for SARS virus [108]	2003	Nature	554
Transmission dynamics and control of severe acute respiratory syndrome [109]	2003	Science	535
Epidemiological determinants of spread of causal agent of severe acute respiratory syndrome in Hong Kong [110]	2003	Lancet	515

^a^ *PNAS* Proceedings of the National Academy of Sciences

### Country analysis

**Fig. 3:**
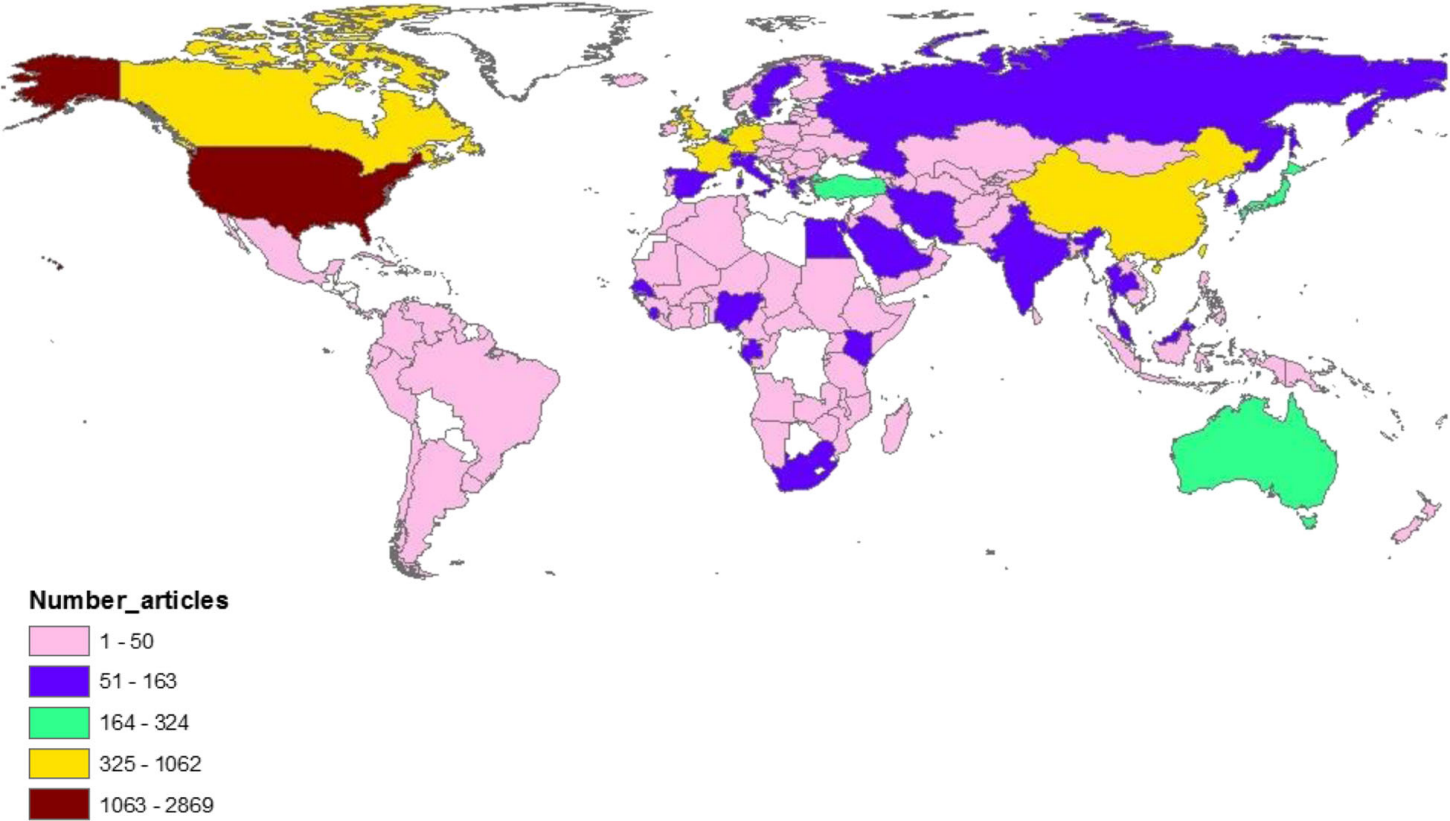
Geographical distribution of publications on the eight emerging pathogens. The map was created using ArcGIS 10.1 program. Regions with no colors in the map have no available data

Researchers from 154 different countries participated in publishing retrieved articles. Table 4 shows a list of countries with a minimum contribution of 100 articles. The list included 23 different countries in North America, Middle East, Europe, Asia, Australia, and Africa. The total number of articles produced by the list of active countries was 6892 (80.0%). The United States of America (USA) ranked first in productivity with a total of 2852 (33.1%) followed by China (*n* = 1,057; 12.3%), Hong Kong (*n* = 548; 6.4%), and Germany (*n* = 608; 7.1%). Geographical distribution of worldwide publications on the top eight emerging pathogens was mapped using ArcGIS 10.1 with darker colors indicative of higher productivity (Fig. 3).

**Table 4:** List of countries with a minimum contribution of 100 documents

Country	Frequency^a^	% *N* = 8619	SCP	%	MCP	%	TC	C/A	*h*-index
USA	2852	33.1	1499	52.6	1353	47.4	111552	39.1	145
China	1057	12.3	604	57.1	453	42.9	23153	21.9	67
Germany	608	7.1	203	33.4	405	66.6	28217	46.4	77
Hong Kong	548	6.4	326	59.5	222	40.5	26917	49.1	71
Canada	527	6.1	202	38.3	325	61.7	21809	41.4	75
France	521	6.0	170	32.6	351	67.4	18658	35.8	68
UK	470	5.5	132	28.1	338	71.9	14704	31.3	60
Japan	324	3.8	121	37.3	203	62.7	8309	25.6	48
Turkey	306	3.6	269	87.9	37	12.1	4375	14.3	30
Taiwan	285	3.3	228	80.0	57	20.0	7161	25.1	39
Singapore	255	3.0	142	55.7	113	44.3	9270	36.4	43
Netherlands	209	2.4	63	30.1	146	69.9	12989	62.1	50
Australia	195	2.3	43	22.1	152	77.9	6262	32.1	43
South Africa	160	1.9	47	29.4	113	70.6	6219	38.9	41
Switzerland	160	1.9	21	13.1	139	86.9	8659	54.1	44
Spain	150	1.7	61	40.7	89	59.3	3883	25.9	33
Saudi Arabia	142	1.6	51	35.9	91	64.1	4733	33.3	38
Italy	140	1.6	54	38.6	86	61.4	2930	20.9	29
India	123	1.4	74	60.2	49	39.8	1207	9.8	18
Belgium	109	1.3	18	16.5	91	83.5	4054	37.2	34
Sweden	104	1.2	33	31.7	71	68.3	2498	24.0	31
Iran	102	1.2	84	82.4	18	17.6	1264	12.4	19
Malaysia	100	1.2	58	58.0	42	42.0	4395	44.0	30

*TC* total citations, *C/A* citation per article, *h-index* Hirsch index, *USA* United States of America, *UK* United Kingdom, *SCP* single country publications, *MCP* multiple country publications

^a^When productivity of each country was calculated alone the total number exceeds the number of retrieved articles. However, when productivity of all countries was dealt with collectively, the total number will be lesser than that presented in the table. The collaboration between countries created some percentage of overlap and therefore certain number of similar countries were counted twice for collaborating countries

**Fig. 4:**
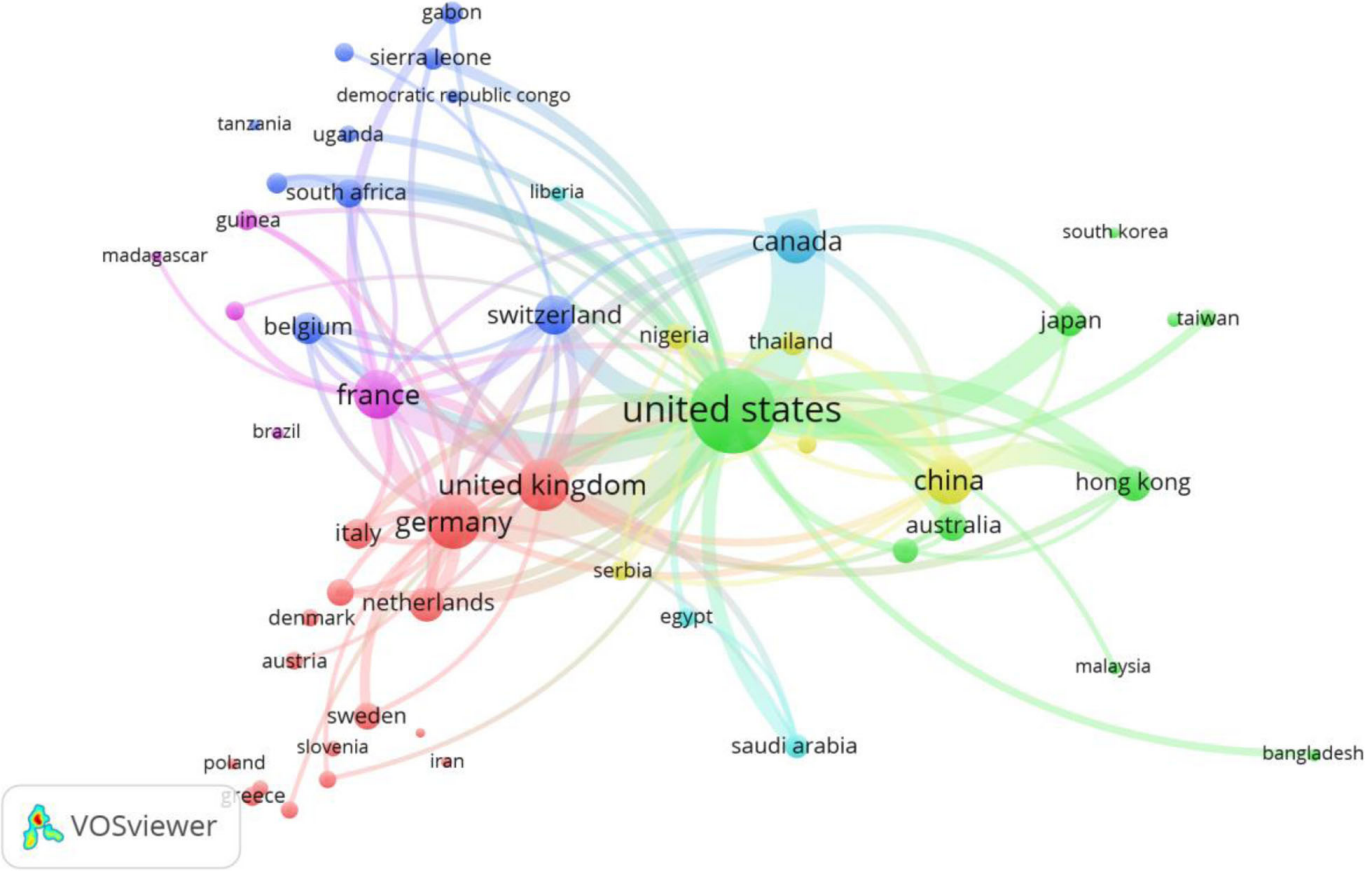
Network visualization of inter-country collaborations among countries with minimum of 20 publications on emerging pathogens. Links represent the strength of collaboration

International collaboration ranged from 12.1 to 86.9%. Turkey had the lowest percentage (12.1%) of articles with international authors while Switzerland had the highest percentage (86.9%) of articles with international authors. Only two countries (Turkey and Iran) had less than 20% international collaboration. There was a significant correlation (Pearson correlation *r* = 0.52; *p* = 0.01) between percentage of international collaboration and number of citation per article but not with *h*-index. Visualization of international collaboration was created using VOSviewer technique. In the network visualization map, the strength of collaboration between countries is expressed by the thickness of the line between any two countries. Figure 4 shows inter-country collaboration between various developed and developing countries. The thickness of the connecting lines represents the extent of collaboration between any two countries.

### Institutions/organizations

Sixteen intuitions/organizations made a contribution of a minimum of 100 publications (Table 5). The total number of documents published by these active institutions was 3083 (35.8%). Eight active intuitions are in northern America (USA and Canada), three are in Hong Kong/China, two in Germany, one in France, one in Japan, and one is an international organization (WHO). The *Centers for Disease Control and Prevention* (CDC) had the highest productivity of 344 (5.5%) articles followed by the *University of Hong Kong* with 305 (4.5%) documents. World Health Organization ranked 12^th^ with 135 (1.6%) documents. However, publications by WHO had the highest citations per article (70.3) followed by those published by *University of Hong Kong* (60.4) and CDC (60.2). The CDC had the highest (87) *h*-index followed by *U.S. Army Medical Research Institute of Infectious Diseases* (75) and *The University of Hong Kong* (63).

**Table 5:** List of institutions/organizations with a minimum contribution of 100 documents

Institution/Organization	Frequency	% *N* = 8619	TC	C/A	*h*-index	Affiliation
Centers for Disease Control and Prevention (CDC)	422	4.9	25410	60.2	87	USA
The University of Hong Kong	305	3.5	18425	60.4	63	Hong Kong
U.S. Army Medical Research Institute of Infectious Diseases	297	3.4	17027	57.3	75	USA
National Institutes of Health, Bethesda	209	2.4	7072	33.8	44	USA
UT Medical Branch at Galveston	208	2.4	6937	33.4	44	USA
National Institute of Allergy and Infectious Diseases	200	2.3	10371	51.9	56	USA
Universitat Marburg	182	2.1	10159	55.8	52	Germany
Institut Pasteur, Paris	175	2.0	8639	49.4	44	France
University of Manitoba	165	1.9	7289	44.2	51	Canada
National Microbiology Laboratory	163	1.9	7250	44.5	48	Canada
Prince of Wales Hospital Hong Kong	141	1.6	6600	46.8	40	Hong Kong
Organisation Mondiale de la Sante	135	1.6	9496	70.3	44	WHO
National Institute of Infectious Diseases	125	1.5	2356	18.8	28	Japan
Chinese University of Hong Kong	114	1.3	4601	40.4	33	Hong Kong
Bernhard Nocht Institut fur Tropenmedizin Hamburg	108	1.3	7241	67.0	36	Germany
University of Toronto	100	1.2	5071	50.7	32	Canada

*TC* total citations, *C/A* citation per article, *h-index* Hirsch index, *USA* United States of America, *WHO* World Health Organization

### Journals and authors

Five journals made a contribution of at least 100 articles to studied diseases. Top leading journal was *Journal of Virology* with 572 (6.6%) articles. The journal is published by the *American Society of Microbiology* and has an IF of 4.6. The second ranking journal was *Emerging Infectious Diseases* with 295 (3.4%) publications; published by the CDC and has and IF of 6.99. The third ranking journal was *Journal of Infectious Diseases* with 244 (2.8%) articles; published on behalf of *Infectious Diseases Society of America* and has an IF of 6.3. The fourth ranking journal was *Virology* journal with 194 (2.3%) articles; published by *Elsevier* and has an impact factor of 3.2. The fifth ranking journal was *Plos One* with 146 (1.7%) articles; published by *Public Library of Science*, and has an IF of 3.1.

**Fig. 5:**
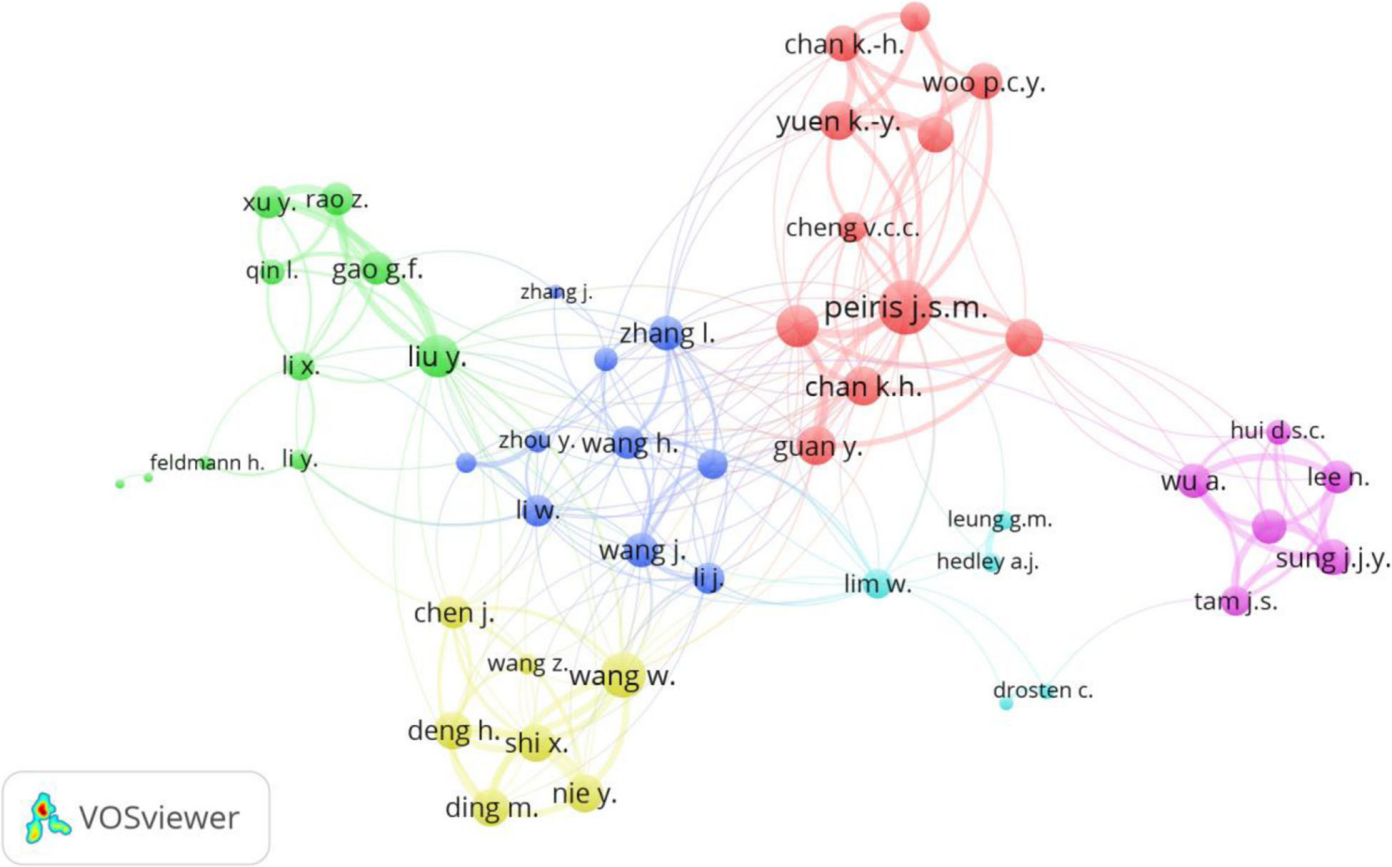
Network visualization map of author collaboration. Cluster of authors having similar cluster color most probably represents a closely related research group

Feldmann, Heinz R. at the *National Institutes of Health, Bethesda, Laboratory of Virology*, was the most productive researcher with 197 (2.3%) articles. Rollin, Pierre Etienne at *CDC, Atlanta, USA* ranked second with 123 (1.4%) articles. Ksiazek, Thomas G. at *Galveston National Laboratory, Galveston, USA* ranked third with 118 (1.4%) articles. Nichol, Stuart T., at the *National Center for Emerging and Zoonotic Infectious Diseases, Atlanta, USA*, ranked fourth with 112 (1.3%) articles. Geisbert, Thomas Thomas, at *UT Medical Branch at Galveston, Department of Microbiology and Immunology, Galveston, USA* ranked fifth with 103 (1.2%) articles. Figure 5 is a visualization map of author collaboration. The map had 6 clusters of names of authors. Each cluster represents a research group working on particular pathogen(s).

### Publication activity on each disease

**Fig. 6:**
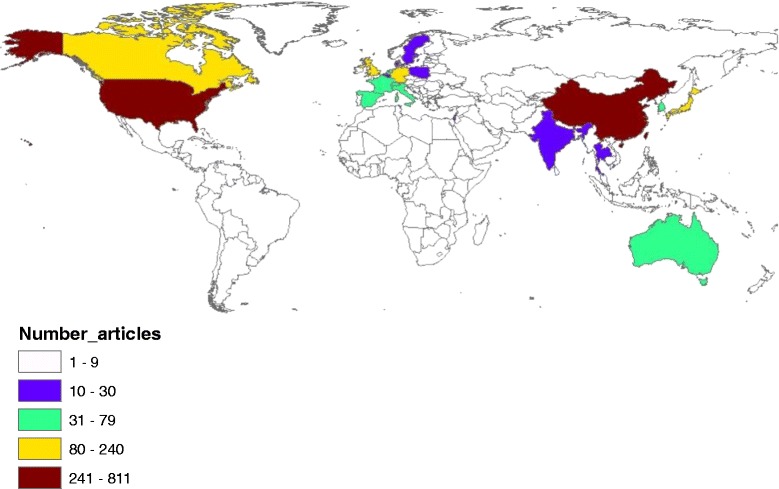
Geographical distribution of publications on SARS. The map was created using ArcGIS 10.1 program. Regions with no colors in the map have no available data

**Fig. 7:**
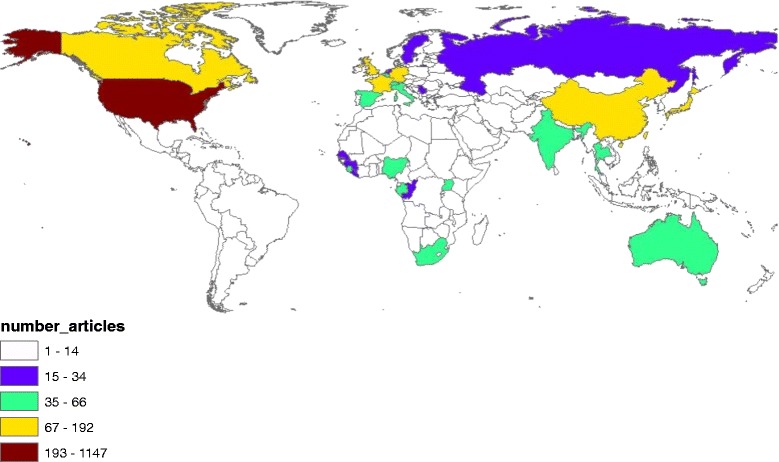
Geographical distribution of publications on Ebola. The map was created using ArcGIS 10.1 program. Regions with no colors in the map have no available data

**Fig. 8:**
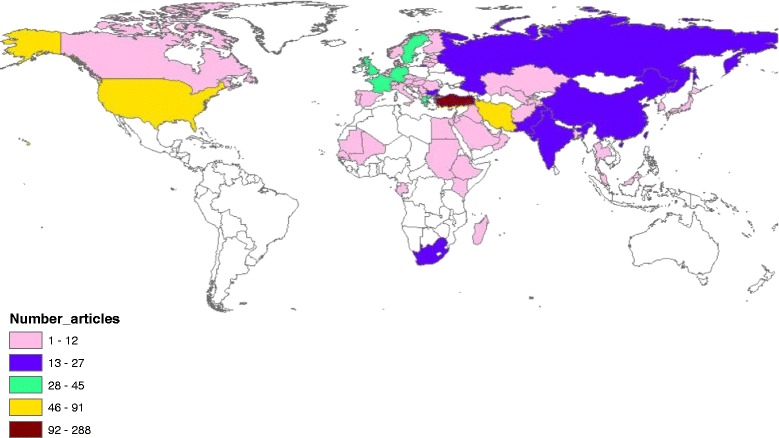
Geographical distribution of publications on Ebola. The map was created using ArcGIS 10.1 program. Regions with no colors in the map have no available data

**Fig. 9:**
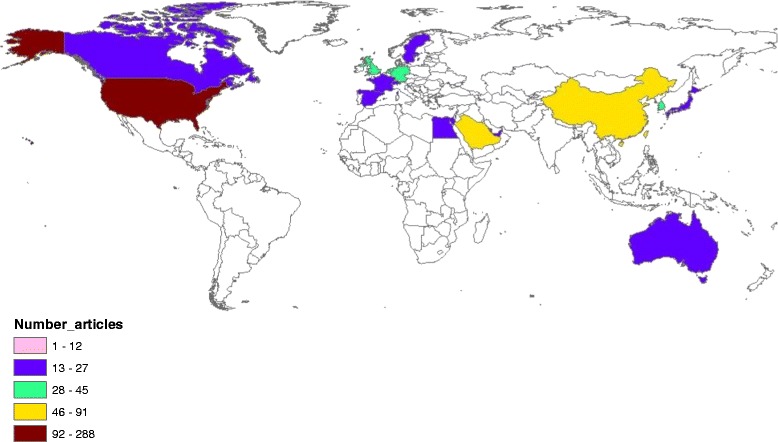
Geographical distribution of publications on MERS. The map was created using ArcGIS 10.1 program. Regions with no colors in the map have no available data

**Fig. 10:**
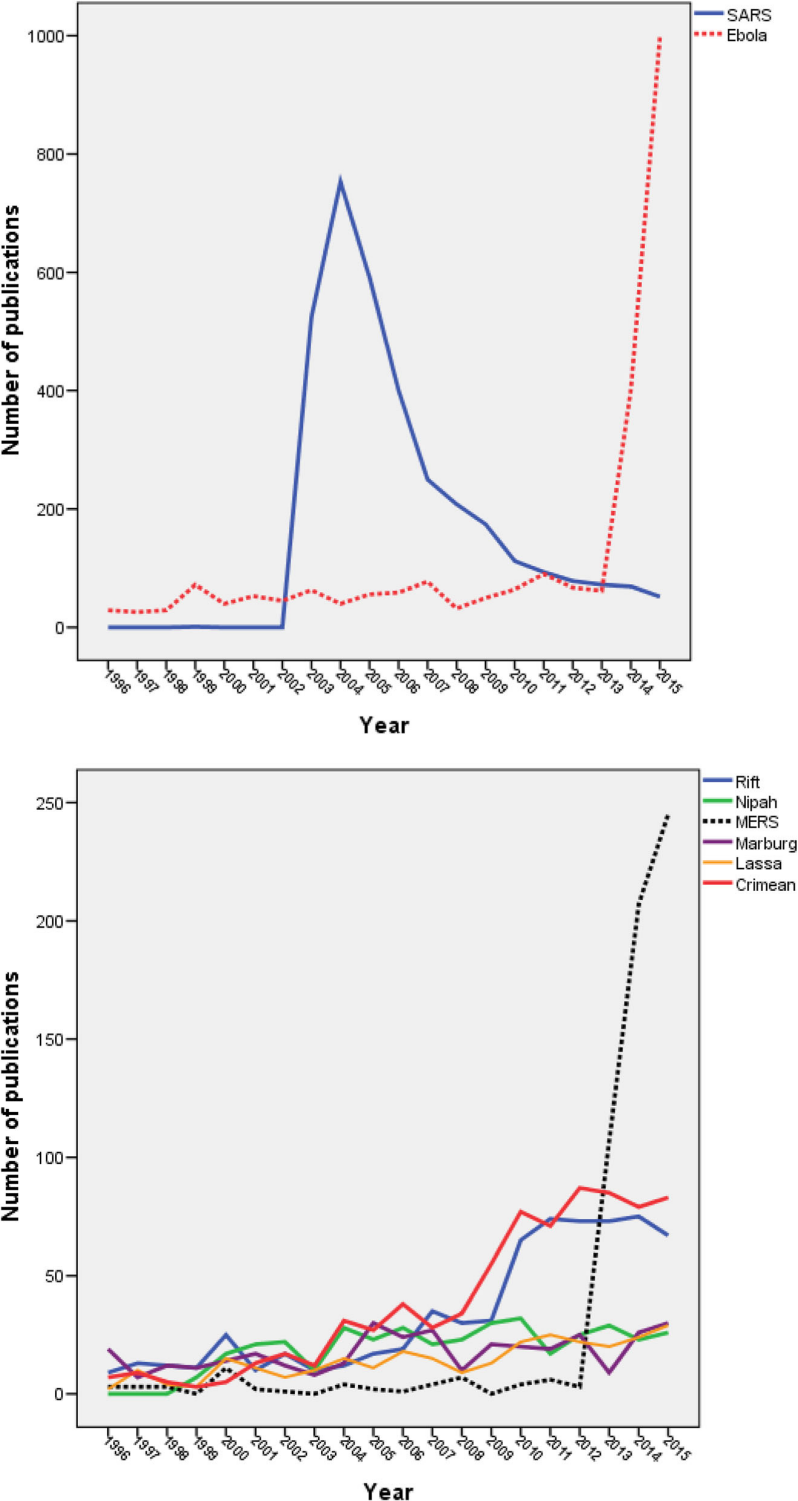
**a** Growth of publication on Ebola and SARS (1996–2015). **b** Growth of publications on “Crimean – Congo, Marburg, Lassa fever, Middle East respiratory syndrome (MERS), Nipah, and Rift Valley fever” (1996–2015)

Table 6 shows the number of retrieved articles for each type of disease. Due to the presence of articles that might have discussed more than one pathogen/infectious disease at the same time, the total percentages exceeded 100%. Publications on SARS (3379; 39.2%) ranked first in quantity followed by those on Ebola (2355; 27.3%) and Crimean-Congo (766; 8.9%). Geographical distribution of research publications on SARS, Ebola, Crimean – Congo, and MERS were mapped and presented in Figs. 6, 7, 8 and 9. The annual growth of publications showed that publications on SARS exhibited a sharp peak in 2003, publications on Ebola exhibited a sharp peak in 2014, and publications on MERS exhibited a clear rise starting from 2012 (Fig. 10a and b).

**Table 6:** Number of publications on each disease

Rank	Disease	Frequency	%	*h*-index
1^st^	SARS	3379	39.2	115
2^nd^	Ebola	2355	27.3	120
3^rd^	Crimean – Congo	766	8.9	54
4^th^	Rift valley fever	678	7.9	61
5^th^	MERS	613	7.1	51
6^th^	Nipah	382	4.4	63
7^th^	Marburg	354	4.1	55
8^th^	Lassa	285	3.3	47

*SARS* Severe acute respiratory syndrome, *MERS* Middle East respiratory syndrome, *h-index* Hirsch index

Due to overlap, total percentage exceeded 100%

Country analysis of publications on each disease is shown in Table 7. The USA ranked first in productivity in research pertaining to Mraburg, Ebola, Rift valley fever, Nipah, MERS, and Lassa. However, China ranked first in SARS while Turkey ranked first in Crimean-Congo fever. For SARS virus, half of the top 10 list were Asian countries while for Nipah virus, four Asian countries appeared in the top 10 list; Malaysia, Bangladesh, Japan, and Singapore. The USA, the UK, and Germany appeared in the top 10 productive list for all diseases. China and/or Hong Kong were in the top 10 productive list for Ebola, MERS, and SARS. Analysis of *h*-index of publications pertaining to each disease showed that publications on Ebola (120) had the highest *h*-index followed by SARS (115), Nipah (63) and rift valley fever (61).

**Table 7:** Top 10 productive countries for each pathogen/infectious disease

SARS	Frequency	% *N* = 3379	Crimean-Congo	Frequency	% *N* = 766	Marburg	Frequency	% *N* = 354
China	811	24.0	Turkey	288	37.6	USA	154	43.5
USA	786	23.3	Iran	91	11.9	Germany	65	18.4
Hong Kong	499	14.8	USA	91	11.9	Canada	29	8.2
Taiwan	276	8.2	UK	45	5.9	Japan	23	6.5
Canada	240	7.1	Germany	41	5.4	France	20	5.6
Singapore	211	6.2	Greece	41	5.4	UK	18	5.1
Germany	167	4.9	France	36	4.7	Belgium	13	3.7
UK	131	3.9	Sweden	35	4.6	Switzerland	13	3.7
Japan	127	3.8	Russian Fed.	27	3.5	South Africa	12	3.4
Netherlands	79	2.3	Bulgaria	24	3.1	Congo	11	3.1
Ebola	Frequency	% *N* = 2355	Rift valley fever	Frequency	% *N* = 678	MERS	Frequency	% *N* = 613
USA	1147	48.7	USA	263	38.8	USA	201	32.8
Canada	192	8.2	France	124	18.3	Saudi Arabia	96	15.7
France	192	8.2	South Africa	75	11.1	China	82	13.4
Germany	186	7.9	Kenya	68	10.0	UK	55	9.0
UK	160	6.8	Senegal	46	6.8	Germany	54	8.8
China	122	5.2	UK	41	6.0	Hong Kong	45	7.3
Japan	113	4.8	Saudi Arabia	31	4.6	Netherlands	44	7.2
Switzerland	66	2.8	Egypt	28	4.1	South Korea	37	6.0
Nigeria	58	2.5	Germany	28	4.1	France	27	4.4
India	56	2.4	Netherlands	27	4.0	Japan	23	3.8
Nipah	Frequency	% *N* = 382	Lassa	Frequency	% *N* = 285			
USA	186	48.7	USA	123	43.2			
Malaysia	88	23.0	Germany	74	26.0			
Australia	66	17.3	France	34	11.9			
Bangladesh	29	7.6	Nigeria	32	11.2			
France	25	6.5	Sierra Leone	19	6.7			
Canada	23	6.0	Guinea	17	6.0			
Japan	19	5.0	Canada	15	5.3			
Germany	17	4.5	UK	12	4.2			
Singapore	16	4.2	Netherlands	10	3.5			
UK	15	3.9	Belgium	9	3.2			
			Japan	9	3.2			

*SARS* Severe acute respiratory syndrome, *MERS* Middle East respiratory

### Publications on vaccine development

**Fig. 11:**
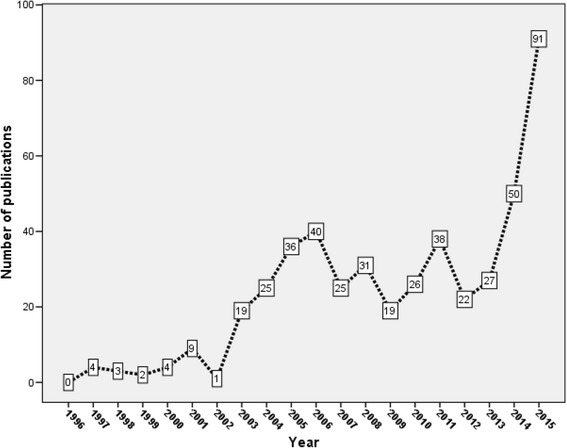
Growth of publications on vaccine research on emerging pathogens (1996–2015)

Four hundred seventy-two publications were related to vaccine development. Research activity on vaccine development showed similar trend to overall research activity on the top eight emerging disease (Fig. 11). As expected *Vaccine* journal (68, 14.4%) ranked first in productivity followed by *Journal of Virology* (40, 8.5%). The USA was the most productive country in this field with 254 (53.8%) followed distantly by China (70; 14.8%) and Canada (54; 11.4%). Professor Feldmann H. (36; 7.6%) was the most prolific author in this field. Top 20 cited articles on vaccines against studied pathogens/infectious diseases are shown in Table 8. Development of a vaccine against Ebola, SARS, Nipah, or Lassa was the main focus of vaccine – related studies. Ten articles in the top 20 list were about Ebola, five were about SARS, two were about Marburg, one was about Nipah, one about Lassa fever, and one article was about both Ebola and Marburg viruses.

**Table 8:** Top 20 cited articles on vaccine – related publication on studied diseases

Title	Year	Journal	Number of citations
Development of a preventive vaccine for Ebola virus infection in primates [111]	2000	Nature	490
Accelerated vaccination for Ebola virus haemorrhagic fever in non-human primates [112]	2003	Nature	336
Live attenuated recombinant vaccine protects nonhuman primates against Ebola and Marburg viruses [113]	2005	Nature Medicine	320
A DNA vaccine induces SARS coronavirus neutralization and protective immunity in mice [114]	2004	Nature	320
Severe acute respiratory syndrome coronavirus spike protein expressed by attenuated vaccinia virus protectively immunizes mice [115]	2004	PNAS	232
Marburg virus vaccines based upon alphavirus replicons protect guinea pigs and nonhuman primates [116]	1998	Virology	199
Effects of a SARS-associated coronavirus vaccine in monkeys [117]	2003	Lancet	168
Ebola virus: From discovery to vaccine [118]	2003	Nature Reviews Immunology	168
Evaluation in nonhuman primates of vaccines against Ebola virus [119]	2002	Emerging Infectious Diseases	166
Severe acute respiratory syndrome vaccine development: Experiences of vaccination against avian infectious bronchitis coronavirus [120]	2003	Avian Pathology	152
Ebola virus-like particle-based vaccine protects nonhuman primates against lethal Ebola virus challenge [121]	2007	Journal of Infectious Diseases	149
Efficacy and effectiveness of an rVSV-vectored vaccine expressing Ebola surface glycoprotein: interim results from the Guinea ring vaccination cluster-randomised trial [122]	2015	The Lancet	137
DNA vaccines expressing either the GP or NP genes of Ebola virus protect mice from lethal challenge [123]	1998	Virology	136
A DNA vaccine for Ebola virus is safe and immunogenic in a phase I clinical trial [124]	2006	Clinical and Vaccine Immunology	134
Single-injection vaccine protects nonhuman primates against infection with Marburg virus and three species of Ebola virus [125]	2009	Journal of Virology	127
Development of a new vaccine for the prevention of Lassa fever [126]	2005	PLoS Medicine	115
Receptor-binding domain of SARS-CoV spike protein induces highly potent neutralizing antibodies: Implication for developing subunit vaccine [127]	2004	Biochemical and Biophysical Research Communications	115
Correlates of protective immunity for Ebola vaccines: Implications for regulatory approval by the animal rule [128]	2009	Nature Reviews Microbiology	105
Nipah Virus: Vaccination and Passive Protection Studies in a Hamster Model [129]	2004	Journal of Virology	105
Recombinant modified vaccinia virus Ankara expressing the spike glycoprotein of severe acute respiratory syndrome coronavirus induces protective neutralizing antibodies primarily targeting the receptor binding region [130]	2005	Journal of Virology	101

*PNAS* Proceedings of the National Academy of Sciences

## Discussion

This study was carried out to assess worldwide research activity on emerging pathogens expected to cause serious fatal outbreaks in the near future. Several bibliometric studies were carried out and published on infectious diseases in general or on a specific disease such as Ebola [36], SARS [37, 38], and Nipah [39, 40]. However, no bibliometric study was carried out on research activity on a group of viruses suspected of potential outbreaks in the near future. These emerging pathogens need to be looked at as one unit since most of them have similar pathogenic and epidemiologic characteristics.

Our study showed that research activity on emerging pathogens showed an uprising peak in 2003 due to the outbreak of SARS at that time, particularly in Asian countries. Another uprising peak of publications was seen in 2014 due to outbreak of Ebola virus and to a lesser extent the outbreak of MERS-CoV. Between the two peaks of SARS and Ebola, there was a high plateau of research activity that is most probably due to the rise in the number of publications about the remaining five diseases.

International collaboration in research on emerging diseases was high possibly due to spread of these viral infectious outbreaks across borders. Furthermore, the relatively high *h*-index of 173 indicates that research on these diseases is receiving a high number of citations suggestive of importance and large number of readers. A study concluded that the *h-*index can be used to estimate the potential impact of a pathogen and to rank individual pathogens or types of pathogens [41]. In our study, Ebola and SARS had the highest *h*-indices which necessitate prioritizing these two pathogens in planning for the future preventive policies. The finding that Professor Feldmann, R. was the most prolific researcher was confirmed by other bibliometric studies [34].

Infectious diseases like acquired immune deficiency syndrome (AIDS), malaria, and tuberculosis are major infectious diseases affecting millions of people and draining billions of US dollars of research funds [42, 43]. Research activity on malaria, tuberculosis, and AIDS have made some success in controlling the spread of such diseases and in developing potent and effective therapies. For example, the discovery of the effective drug artemisinin has greatly changed the therapeutic approach of malaria and enhanced control and eradication of malaria [44–46]. Actually, the Chinese scientist Tu Youyou, who discovered the drug artemisinin, was awarded Nobel Prize in Medicine in 2015 [47, 48]. In case of the top eight emerging pathogens which are expected to cause serious outbreaks in the near future, no effective therapy is available so far and no preventive measures are being developed to face a sudden worldwide outbreak of these infectious diseases. Calls for strengthening preparedness for Crimean-Congo [49] and MERS-coronavirus [50–52] have been published. The WHO stated that research remains the cornerstone for reversing trends of serious outbreaks of certain viral diseases and that research will improve methods for surveillance, prevention, and control. Unfortunately, the increased funding for AIDS created a shortage of funding for other infectious diseases [53]. A study that compared research output and citations among three infectious diseases indicated that funding has a positive influence on research output and citations for a particular disease [54].

In most bibliometric studies, the USA, the UK, Germany, and other European countries appeared in the most active list of publications. However, in this study, additional countries in Asia and Africa, and Middle east did appear in the top active list for each disease emphasizing the global threat of such infectious diseases. A bibliometric analysis on infectious diseases reported that USA ranked as top productive country but China is increasing its place among the top five countries [55]. Actually, many countries start to focus their research efforts on infectious diseases as a national health burden [56]. The participation of Asian, African, and Middle eastern countries in research activity pertaining to top eight emerging infectious diseases was clear and prominent. Outbreaks of emerging viral infectious diseases have been commonly reported from many countries in Africa, Asia, and Africa [52, 57–62]. For example, MERS-CoV and Crimean-Congo fever have been reported in more than 20 countries, mostly in Asia, Africa, and Middle East [63–77]. The outbreaks of SARS in Hong Kong and China had a great economic and public health impact [78–80]. Many of these infectious diseases were initially reported in Africa, such as Ebola, Lassa fever, and Rift valley fever [81–86]. The Marburg virus was initially reported in Germany and spread to other neighboring countries and that is why China and Hong Kong did not show in the top productive countries on Marburg disease.

Our study has few limitations that need to be stated. Scopus is a large and comprehensive database but not all journals are indexed in Scopus and therefore, some articles about the studied diseases published in un-indexed journals might be missed. Furthermore, the keywords used might not be 100% accurate although the validity of the search query was tested by manual review of 10% of retrieved articles, false positive and false negative results remain a possibility. The ranking of countries and institutions based on citations did not take into account self-citations which affects the validity of results. These limitations and others are found in most bibliometric studies [71, 87–91]. This study focused only on the top eight emerging infectious diseases expected to cause severe outbreaks in the near future. However, the other three serious infectious diseases which in include Zaika were not included in the analysis. Finally, we should always bear in mind that no database is perfect and even might have some bias by over-representing journals with English language. Therefore, bibliometric results should always be considered with caution [92].

## Conclusions

The number of publications on diseases expected to cause severe outbreaks in the near future showed two clear peaks in the past two decades; one for SARS and one for Ebola. The clear increase in number of publication on the studied diseases during relatively short period of time is an indication of how science and health information flows rapidly across borders to create similar concerns among different countries. Bibliometric methods can be used to prioritize efforts and direct research funds to help control emerging diseases [41]. Although the USA is leading the research on these diseases, the share of Asian, African, and Middle Eastern countries was apparent. International collaboration in research on these diseases was relatively high for most countries. Search for an effective vaccine was clearly strong for Ebola and SARS.

## Abbreviations

MERS: Middle East respiratory Syndrome
SARS: Severe acute respiratory syndrome
WHO: World Health Organization
